# Resurgence of human coronavirus OC43 at a long-term care facility during the coronavirus disease 2019 (COVID-19) pandemic: Outbreak investigation

**DOI:** 10.1017/ash.2023.172

**Published:** 2023-06-05

**Authors:** Koji Ohyama, Hitoshi Honda, Momoko Aoki, Mitsutaka Wakuda, Tomoaki Kitahara, Chisaki Kaede, Yohei Doi, Yuki Uehara

**Affiliations:** 1 Department of Infectious Diseases, Fujita Health University School of Medicine, Toyoake, Aichi, Japan; 2 Social Welfare Corporation IKOI, Kuwana, Mie, Japan; 3 Aoki Memorial Hospital, Kuwana, Mie, Japan; 4 Department of Laboratory Medicine, Fujita Health University School of Medicine, Toyoake, Aichi, Japan; 5 Division of Infectious Diseases, University of Pittsburgh School of Medicine, Pittsburgh, Pennsylvania, United States

## Abstract

The incidence of seasonal infections due to respiratory viruses other than severe acute respiratory coronavirus virus 2 (SARS-CoV-2) has declined due to heightened public infection prevention measures against coronavirus disease 2019 (COVID-19). We describe an outbreak of human coronavirus OC43 infection that occurred at a long-term care facility and whose clinical features were indistinguishable from COVID-19.

In the early phase of the coronavirus disease 2019 (COVID-19) pandemic, public health interventions, such as social distancing, use of face coverings, and stay-at-home orders, were implemented to prevent transmission of severe acute respiratory syndrome coronavirus 2 (SARS-CoV-2). Meanwhile, the incidence of respiratory viral infections due to influenza virus, respiratory syncytial virus (RSV), and other common respiratory viruses have declined substantially.^1,2^ However, as public health interventions against COVID-19 are gradually lifted, a resurgence of respiratory infections due to common non–COVID-19 respiratory viruses has been observed.^3–5^ For instance, influenza and RSV infection have reemerged in the United States in the 2022–2023 winter season to cause a triple epidemic, or “tridemic.”^6^ Although the reasons for the resurgence of seasonal viral infections are not entirely clear, public health interventions to prevent the spread of SARS-CoV-2 may have also limited exposure to other respiratory viruses in the community, leading to diminished immunity, thus increased susceptibility to these viruses among the public.^7,8^

Over the same winter season, an outbreak of human coronavirus OC43 (HCoV-OC43) occurred at a long-term care facility (LTCF) in Japan, which was initially presumed to be caused by SARS-CoV-2 due to indistinguishable clinical presentations. Here, we report our investigation of this unusual outbreak and discuss implications of the resurgence of respiratory viruses other than SARS-CoV-2 during the COVID-19 pandemic.

## Outbreak investigation

An outbreak investigation was conducted at an LTCF in central Japan. The facility has 70 available beds, consisting of 12 four-bed rooms and 22 single rooms on 2 floors. It also contains shared spaces including restrooms, bathrooms, dining rooms, and rehabilitation training rooms. Healthcare personnel were required to wear mask, maintain physical distance from residents whenever possible and avoid unnecessary conversation during mealtime at the shared break room. In January 2023, several residents in the facility simultaneously developed fever and respiratory symptoms. The SARS-CoV-2 rapid antigen test (QuickNavi-COVID19 Ag) was negative for these symptomatic individuals. Because of an increasing number of symptomatic individuals and uncertainty over the cause of the outbreak, infectious disease physicians from a nearby university hospital joined the investigation. Also, 8 days after identification of the index case, nasopharyngeal swab specimens from 3 representative symptomatic LTCF residents were tested using multiplex polymerase chain reaction (PCR) (BIOFIRE Respiratory 2.1 panel). All were positive for HCoV-OC43. Given this finding, the same test was performed the following day on all residents with fever and respiratory symptoms as well as those sharing room with symptomatic residents at the facility.

Ethical approval for this study was granted by the Ethics Committee of Fujita Health University (HM22-479).

## Results

Of the 26 residents who underwent multiplex PCR testing, 22 (84.6%) were positive for HCoV-OC43. Table 1 shows the clinical characteristics of the 22 residents with HCoV-OC43 infection. The median age was 89 years (interquartile range [IQR], 80–95), and 6 (27.3%) were male. All patients were long-term residents with a median length of stay from admission to symptom onset of 920 days (IQR, 363–1,432). The median number of COVID-19 vaccine (BNT162b2 [Pfizer-BioNTech] or mRNA-1273 [Moderna]) doses was 4 (IQR, 3–5). Also, 1 resident (4.5%) and 3 (13.6%) residents had a concurrent positive PCR assays with SARS-CoV-2 and parainfluenza virus type 3 (PIV3), respectively. Common symptoms for individuals with HCoV-OC43 infection were fever (21 of 22; 95.5%) and cough (20 of 22; 90.9%). Most individuals with HCoV-OC43 infection had impaired basic activities of daily living at baseline, with a median Katz index score of 1 (IQR, 0–4).

**Table 1.** Clinical Characteristics of HCoV-OC43 Infection

Characteristics	Patients with HCoV-OC43 Infection (N = 22)
Age, median y (IQR)	89 (80–95)
Sex, male, no. (%)	6 (27.2)
Katz index of activities of daily living (IQR)	1 (0–4)
Length of stay to symptom onset, median d (IQR)	920 (363–1,432)
No. of COVID-19 vaccine (IQR), median doses	4 (3–5)
**Symptoms, no. (%)**	
Fever	21 (95.4)
Cough	20 (90.9)
**Comorbidities, no. (%)**	
Dementia	16 (72.7)
Cardiovascular diseases	10 (45.4)
Cerebrovascular diseases	6 (27.2)
**Coinfection, no. (%)**	
SARS-CoV-2	1 (4.5)
Parainfluenza virus type 3	3 (13.6)
**Outcome, no. (%)**	
Hospitalization	5 (22.7)
Death	0

Notes. IQR, interquartile range; COVID-19, coronavirus disease 2019; SARS-CoV-2, severe acute respiratory syndrome coronavirus 2.

The index resident patient with HCoV-OC43 infection presented with fever and cough, and the diagnosis of HCoV-OC43 infection was made 8 days after the symptom onset. Subsequently, multiple cases of HCoV-OC43 infection were confirmed among the residents of the facility, as well as residents who shared the room with the index case, whereas the route of transmission from the index case to the subsequent cases remains unclear. Since many residents have impaired basic ADL due to underlying cognitive disorders (i.e., dementia), it was challenging to enforce masking during their stay and physical distancing in shared spaces such as the dining room. It is hypothesized that the spread of HCoV-OC43 occurred among residents in the four-bed rooms and those who shared the dining room.

Of the 22 patients with HCoV-OC43 infection, 5 patients required hospitalization due to respiratory distress, 2 of whom had coinfection with PIV3 and 1 with SARS-CoV-2. All residents with HCoV-OC43 infection were alive 30 days after the initial diagnosis, including those who required hospitalization. Two of the hospitalized residents remained in hospital at day 30. One resident who had coinfection with PIV3 was hospitalized because of prolonged symptoms, and the other resident was hospitalized after recovery from HCoV-OC43 infection due to exacerbation of underlying chronic heart failure.

## Discussion

The outbreak investigation of HCoV-OC43 infections that occurred at an LTCF in Japan 3 years into the COVID-19 pandemic highlighted clinical characteristics of the affected residents. Even with our epidemiological investigation, it was challenging to conclude how the index patient contracted HCoV-OC43 infection in the LTCF. However, given the extended stays in the LTCF, we assume that either a visitor or a healthcare worker unknowingly introduced HCoV-OC43 into the facility, resulting in the outbreak among the residents.

Resurgent non–SARS-CoV-2 respiratory viruses may cause outbreaks both in community and healthcare settings, especially among the elderly and populations with underlying illnesses. As social activities have resumed and infection prevention measures against COVID-19 transmission have been relaxed in the community, many countries have experienced resurgence of respiratory viruses other than SARS-CoV-2.^3–5^ In the present outbreak, we identified 4 patients with coinfection, 1 patient with SARS-CoV-2, and 3 patients with PIV3, suggesting that a number of respiratory viruses were circulating during the period. Although the reasons for the resurgence of non–SARS-CoV-2 respiratory viruses remain unclear, it might be associated with waning immunity against these viruses due to reduced exposure over the course of the COVID-19 pandemic due to heightened infection prevention measures.

One study reported lower RSV antibody levels and diminished neutralization potency of the serum among women of childbearing age and infants compared to prepandemic levels.^7^ Similar to RSV, HCoV-OC43 is a seasonal respiratory virus that commonly causes upper respiratory tract infection, which is usually self-limiting in adults. However, as seen in the current outbreak, some patients at high risk of severe illness developed respiratory distress and required hospitalization and experienced exacerbation of their underlying conditions, leading to a prolonged hospital stay. Notably, 3 of 4 patients with coinfection detected by multiple PCR required hospitalization, suggesting potential additive pathogenicity due to coinfection. Previous reports have suggested association of concurrent respiratory viral infections with increased severity of respiratory distress and poor prognosis.^9,10^ Furthermore, most residents had been vaccinated with booster doses for SARS-CoV-2, and the consequent mild illness made it challenging to differentiate it from other respiratory viral infections presenting with similar symptoms and severity of disease. Also, the use of a syndromic diagnostic test that simultaneously identified multiple respiratory viruses allowed us to rapidly determine the causative viruses involved in the outbreak as well as their spaciotemporal distribution, which was very helpful in managing the outbreak.

This study had several limitations. Whether the detected HCoV-OC43 had the same genotype was unknown because genotypic testing was not performed. Also, PCR testing was only performed for patients located on 2 floors affected by the outbreak, and a facility-wide investigation was not conducted because of time constraints and limited resources.

In conclusion, we experienced an outbreak of HCoV-OC43 infection at an LTCF that mimicked a typical outbreak of COVID-19. The use of multiplex PCR has become increasingly important to identify causative respiratory pathogens in the COVID-19 pandemic era. The findings highlight the importance of awareness that non–SARS-CoV-2 respiratory viral infections may cause institutional outbreaks as easing of public infection prevention measures creates opportunities for transmission in the community after a 3-year hiatus.
